# Reinterpretation of Report of Tetrataenite in Bulk Alloy Castings

**DOI:** 10.1002/advs.202408796

**Published:** 2024-12-16

**Authors:** Owain S. Houghton, James C. Loudon, Alison C. Twitchett‐Harrison, Nikolaos T. Panagiotopoulos, Giulio I. Lampronti, Miguel B. Costa, Richard J. Harrison, A. Lindsay Greer

**Affiliations:** ^1^ Department of Materials Science and Metallurgy University of Cambridge 27 Charles Babbage Road Cambridge CB3 0FS UK; ^2^ Department of Earth Sciences University of Cambridge Downing Street Cambridge CB2 3EQ UK; ^3^ WPI Advanced Institute for Materials Research Tohoku University Sendai 980–8577 Japan

**Keywords:** electron diffraction1, magnetic hysteresis, order‐disorder, rare‐earth‐free permanent magnet, tetrataenite

## Abstract

After the publication of “Direct formation of hard‐magnetic tetrataenite in bulk alloy castings” Ivanov et al., *Advanced Science* 10 (2022) 2204315, the authors identified a potential misinterpretation of the experimental data. Further work confirms that the original conclusions cannot be supported, and accordingly the paper is retracted. X‐ray diffraction shows the presence of (Fe,Ni)_3_P, giving additional Bragg peaks that were previously attributed to tetrataenite. Extra Bragg reflections observed in transmission electron diffraction, and earlier considered to be superlattice reflections from tetrataenite, instead result from oxidation of the sample surface during preparation. Measurements of magnetic hysteresis show no regions of high coercivity of the kind expected when tetrataenite is present. It is concluded that as‐cast bulk samples of Fe‐Ni‐P‐(C) show no evidence of tetrataenite on any length scale. Nevertheless, the addition of phosphorus should remain of interest in ongoing efforts to manufacture tetrataenite.

## Graphical abstract

After publication, the authors of *Advanced Science* 10 (2022) 2204315 identified a potential misinterpretation of their data. This comment accompanies retraction of that paper, and reports further characterization of cast Fe‐Ni‐P‐(C) alloys using X‐ray diffraction, electron diffraction and magnetometry. There is no evidence of the hard‐magnetic phase tetrataenite; the reasons for the original supposition of its presence are explored.

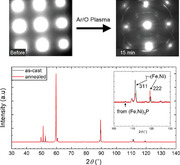

## 1 Introduction

In recent work,^[^ ^1^ ^]^ now retracted, it was suggested that tetrataenite (Strukturbericht L1_0_), L1_0_ FeNi, can be produced as the majority phase in bulk castings of Fe‐Ni‐P‐(C) alloys, and that the presence of phosphorus can accelerate the chemical ordering needed to form tetrataenite (by perhaps up to 10 orders of magnitude). Bulk casting would clearly be an attractive low‐cost production method, motivating further investigation of how alloy composition, in particular phosphorus content, could affect the formation of tetrataenite and the magnetic properties of the samples. The present study reinterprets the data in ref. [1] presents new data, and accompanies the retraction of ref. [1].

**Figure 1:**
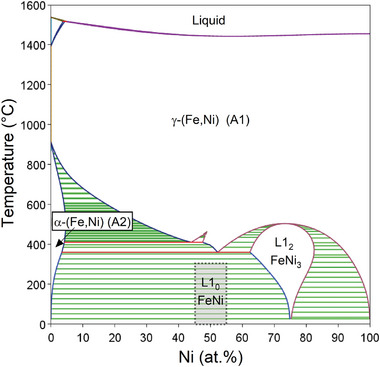
The Fe‐Ni phase diagram. This is computed from experimental data in Thermo‐Calc (SSOL5 database). The shaded regions are two‐phase. Tetrataenite,L1_0_ FeNi, is excluded from the calculations, but its anticipated position^[^ ^2^ ^]^ is overlaid.

The phases of potential interest in the binary Fe‐Ni system are indicated in **Figure** **1**. Across almost the entire composition range, the primary phase expected to form from the melt is the cubic close‐packed (Strukturbericht A1) solid solution, γ‐(Fe,Ni), in which the Fe and Ni atoms are arranged randomly on the atomic sites. When found in meteorites, this phase is termed *taenite*. Around 75 at.% Ni, ordering of Fe and Ni on the sites gives the phase L1_2_ FeNi_3_.^[^ ^3^ ^]^ Chemically ordered tetrataenite is not generally indicated on the equilibrium diagram.^[^ ^4^ ^]^ In iron‐rich compositions, precipitation of body‐centered cubic (Strukturbericht A2) α‐(Fe,Ni) can be expected, and this is found in meteorites as *kamacite*.

The original study^[^ ^1^ ^]^ examined the effects of phosphorus additions of up to 13 at.%. It was shown that the primary Fe‐Ni phase in their cast alloys contained less than 1 at.% P, the remainder being incorporated into (Fe,Ni)_3_P. Computer coupling of phase diagrams and thermochemistry (CALPHAD) calculations in the present work show that the amount of phosphide in (Fe_50_Ni_50_)_1‒_ *_x_* P*_x_* (at.%) rises linearly with P content, reaching a mole fraction of 0.4 at 10 at.% P.

## 2 Results

### 2.1 X‐Ray Diffraction

The pseudo‐cubic unit‐cell of tetrataenite has a tetragonal distortion (δ*a*/*a*) of roughly 0.7% relative to cubic taenite with lattice parameter *a*. This distortion implies that all *hkl* Bragg peaks (where *l* ≠ 0) for γ‐(Fe,Ni) should split upon ordering to form tetrataenite. As in the original study,^[^ ^1^ ^]^ we used X‐ray diffraction (XRD, CoKα) to characterize as‐cast and annealed samples of Fe_50_Ni_30_P_13_C_7_ (at.%). Earlier, for the as‐cast sample, a high‐angle peak was attributed to reflections from the {311} planes of γ‐(Fe,Ni), and the partial splitting of this peak was taken as evidence that ordering to tetrataenite had occurred during casting.^[^ ^1^ ^]^ In the annealed sample, it appeared that this peak had migrated to somewhat higher diffraction angle 2*θ*, and it was no longer split. This apparently irreversible disordering upon annealing is consistent with studies of tetrataenite in meteoritic samples.^[^ ^5^ ^]^

**Figure 2:**
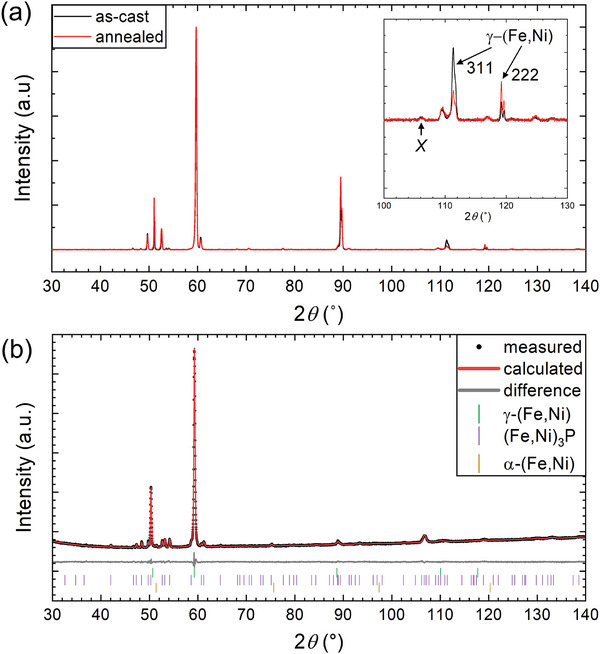
X‐ray diffractogram of (Fe_55_Ni_45_)_94_P_6_. (a) Traces before and after annealing at 1073 K for 1 h. (Inset) The CoKα source is not monochromatic: the high‐angle shoulder on the 311 peak and the splitting of the 222 peak are due to irradiation by both Kα_1_ and Kα_2_ X‐rays. The peak labeled *X* is attributed to (Fe,Ni)_3_P. A similar peak observed in the original study^[^ ^1^ ^]^ was taken to be the 311 peak of γ‐(Fe,Ni) and to be partially split, indicating some ordering to tetrataenite. (b) Rietveld refinement giving a close fit to the diffractogram of an as‐cast sample, and showing assignment of the peaks to three phases: γ‐(Fe,Ni), (Fe,Ni)_3_P, and α‐(Fe,Ni).

In the present work, the composition (Fe_55_Ni_45_)_94_P_6_ is chosen for study because it has a lower volume fraction of metal‐metalloid compounds in the alloy. Both as‐cast and annealed samples show two peaks in the 2*θ* range around the expected 311 peak from γ‐(Fe,Ni) (**Figure** **2**a, and inset). Bragg peaks for (Fe,Ni)_3_P occur in the same 2θ range, and therefore reliable identification of the constituent phases requires a full‐pattern refinement. Rietveld refinement shows that the three‐phase mixture of α‐(Fe,Ni) (A2, *a* = 2.87 Å), γ‐(Fe,Ni) (A1, *a* = 3.62 Å) and (Fe,Ni)_3_P (D0_e_, *a* = 9.08 Å, *c* = 4.47 Å) provides an excellent fit to the full diffractogram (Figure 2b). The introduction of L1_0_ tetrataenite fails to significantly improve the fitting, which suggests that the volume fraction of this phase, if present at all, is less than 1%. A good fitting cannot be achieved without including the pattern for (Fe,Ni)_3_P in the refinement.

The higher‐angle of the two peaks (at 2*θ* ≈ 111.4°, Figure 2a) is similar to that seen for the annealed sample in the original work, and can be reliably identified as the 311 peak of γ‐(Fe,Ni). This indexing is consistent with the angular position of the 222 peak of γ‐(Fe,Ni), which is near 2*θ* = 119.4° (Figure 2a). The lower‐angle peak (2*θ* = 109.6°, labeled *X* in Figure 2a inset) is most likely the 642 peak for (Fe,Ni)_3_P.

From Figure 2a, there appear to be conflicting effects of annealing: the 311 peak suggests that the volume fraction of γ‐(Fe,Ni) decreases as a result of annealing, while the 222 peak suggests the reverse. Our interpretation is that this is a consequence of the coarse microstructure in the as‐cast and annealed samples of (Fe_55_Ni_45_)_94_P_6_. With a typical grain diameter only an order of magnitude smaller than that of the region irradiated in XRD, too few grains are irradiated to ensure a ‘powder‐like’ diffraction pattern, and thus relative peak intensities are influenced by local texture (preferred crystallographic orientation) and by exact positioning of the sample in the X‐ray beam. These issues are relevant also for interpreting the earlier results.^[^ ^1^ ^]^

In the present work, the as‐cast sample of (Fe_55_Ni_45_)_94_P_6_ was annealed for 1 h at 1073 K (i.e., well above the reported order‐disorder temperature of tetrataenite). If a split 311 peak for γ‐(Fe,Ni) in the as‐cast sample were due to tetrataenite, the disordering caused by the annealing should remove the splitting, giving a single peak. Instead, the peak shape remains the same (Figure 2a).

In the original work,^[^ ^1^ ^]^ a partially split peak was observed roughly where *X* is in Figure 2a inset. This was interpreted as an overlap of 103 and 211 peaks, according to the conventional body‐centered tetragonal unit cell of tetrataenite, but we now attribute this to (Fe,Ni)_3_P. The XRD results are thus now re‐interpreted: in the original part of the study, the as‐cast sample shows phosphide, but no 311 peak of γ‐(Fe,Ni): the annealed sample shows the 311 peak but no phosphide. This variation is assumed to be due to the coarse microstructure (as noted above). This reinterpretation removes the need to explain the unexpected shift in overall diffraction angle that appears to accompany disordering in the earlier work.^[^ ^1^ ^]^

### 2.2 Transmission Electron Diffraction

**Figure 3:**
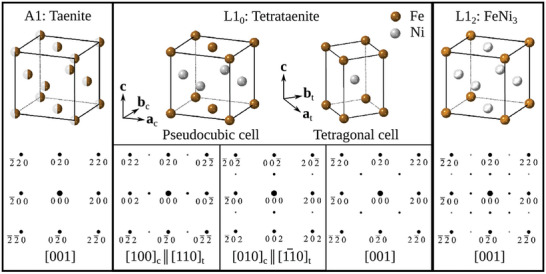
Unit‐cells and simulated electron‐diffraction patterns for phases of Fe‐Ni. The disordered A1 phase shows a diffraction pattern with the systematic absences expected for its face‐centered cubic lattice. Ordering of Fe and Ni atoms (to give L1_0_ or L1_2_ structure) leads to fewer systematically absent reflections (i.e., appearance of superlattice reflections).

Although tetrataenite is not detectable in the present XRD results, it could still be present locally in the as‐cast samples. Indeed, in meteorites, tetrataenite is localized as fine (10–150 nm^[^ ^6^ ^]^) particles dispersed within the *cloudy zone*. Furthermore, the fine microstructure in this zone is necessary to achieve a high‐coercivity magnet.^[^ ^6^, ^7^ ^]^ Thus, finely dispersed regions of L1_0_ FeNi in the casting would be of both scientific and technological interest. To reliably identify L1_0_ FeNi, it is necessary to distinguish it from the chemically disordered taenite and the ordered L1_2_ FeNi_3_ (Figure 1). These structures and their associated electron diffraction patterns are summarized in **Figure** **3**.

The original study^[^ ^1^ ^]^ examined several as‐cast Fe‐Ni‐P‐(C) alloys, and it was considered that the primary‐phase dendrites (easily observed in optical metallography) might consist mainly of tetrataenite. Detailed transmission electron microscopy (TEM) study of Fe‐Ni dendrites in as‐cast Fe_55_Ni_35_P_6.5_C_3.5_ included [001] zone‐axis electron‐diffraction patterns that appeared to show superlattice 110‐type reflections. The thin foils were subjected to cleaning in an Ar/O plasma for 1 min; a process used to remove carbonaceous contaminants by oxidation.

**Figure 4:**
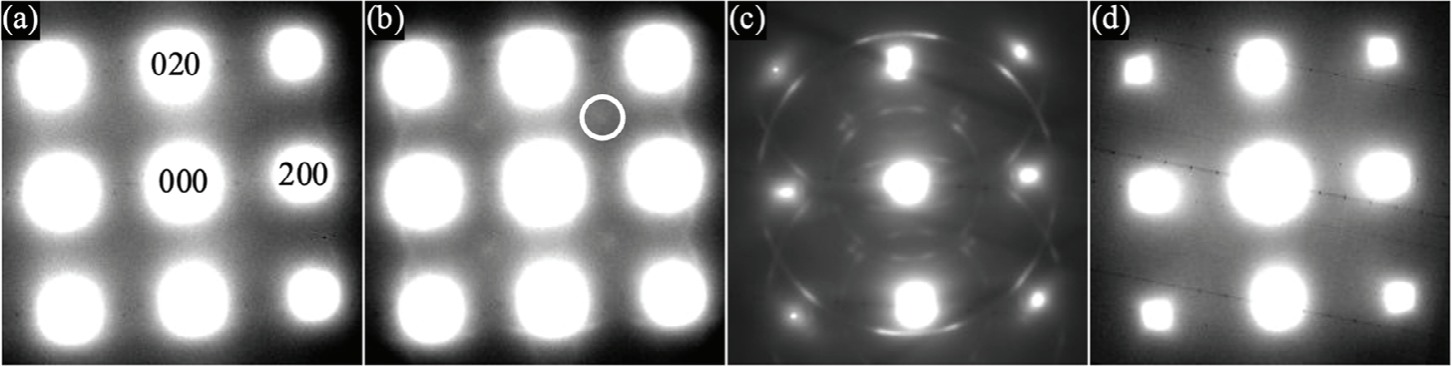
Electron diffraction patterns from the dendritic primary phase in cast Fe_55_Ni_35_P_6.5_C_3.5_. These are on the [001] zone axis and taken from a circular region 750 nm in diameter in a single thin foil. a) From the thin foil before plasma‐cleaning. b) From the same region after plasma‐cleaning for 1 min. The circle highlights one of the new spots of increased intensity that appear near 110‐type positions. c) After plasma‐cleaning for 15 min and heating the sample to 771 K. d) At room temperature after further heating to 880 K. (The faint black lines are an artefact of the camera shuttering).

In the present work, we prepared a fresh thin foil from the same as‐cast rod and initially examined it without any plasma cleaning. The newly obtained electron‐diffraction patterns (on the cubic [001] zone axis) do not show any superlattice reflections (**Figure** **4**a). The thin foil was subsequently plasma‐cleaned, and the diffraction pattern then obtained (Figure 4b), resembles the results in the original work^[^ ^1^ ^]^: patches of increased intensity are visible near 110‐type spots and there are diffuse rings around the 200‐type spots. The rings are attributable to an oxide coating that forms on the foil surface during exposure to the Ar/O plasma. The foil was subjected to this “cleaning” plasma for a further 15 min, but no further changes in the diffraction pattern were observed. In the present work, the intensity at the 110‐type positions is now re‐interpreted as arising at the intersection of the diffuse rings (due to oxidation during plasma‐cleaning), rather than from superlattice reflections of tetrataenite.

The thin foil was then heated in situ to 771 K after which the 110‐type pseudo reflections and additional rings are sharper and more intense (Figure 4c). An inner ring of reflections can also be seen, indicating higher crystallinity in the oxide coating. Three sets of extra spots can be distinguished, consistent (**Table** **1**) with the 220‐, 311‐, and 440‐type reflections of Fe_3_O_4_ (*magnetite*, space group $\mathrm{Fd}\bar{3}m$, with lattice parameter 8.394 Å^[^ ^8^ ^]^) and Fe_2_NiO_4_ (*trevorite*, space group $\mathrm{Fd}\bar{3}m$, with lattice parameter 8.338 Å^[^ ^9^ ^]^). It is consistent with the work of Reuter et al.^[^ ^10^ ^]^ that we observe these reflections of Fe_3_O_4_, but not any 400‐ or 511‐type spots. This implies that the oxide forming on the thin foil has a preferred orientation, with {$1\bar{12}$} planes of Fe_3_O_4_ parallel to {001} of γ‐(Fe‐Ni).^[^ ^10^ ^]^

**Table 1:** Wavenumbers observed in the electron diffraction patterns and the corresponding tabulated values for Fe_3_O_4_ and Fe_2_NiO_4_ (labeled with the {*hkl*} planes assigned to those reflections).

Measured [Å^‒1^]	Fe_3_O_4_ [Å^‒1^]	Fe_2_NiO_4_ [Å^‒1^]
0.341 ± 0.005	0.337 {220}	0.339 {220}
0.391 ± 0.005	0.395 {311}	0.398 {311}
0.677 ± 0.005	0.674 {440}	0.678 {440}

Additional features in Figure 4b,c are attributed to multiple scattering of the electron beam; the same three sets of reflections are repeated, centered not just on 000 but also on the 200‐ and 220‐type spots. When the sample is heated further, the extra reflections disappear by 880 K (Figure 4d). These reflections do not reappear after the sample is cooled to room temperature.

From measurements of the spacing between two perpendicular 200‐type spots in diffraction patterns on the [110] zone axis in the tetragonal setting (Figure 4 in ref. [1]), that earlier work reported a difference in the measured *c* and *a* lattice parameters consistent with the known tetragonality of meteoritic L1_0_ FeNi. In the present work, however, we found that the diffraction pattern could be distorted by variation in the objective astigmatism, giving *c*/*a* ratios similar to those reported in ref. [1].

The apparent superlattice reflections in Figure 4b (and in Figure 5 in ref. [1]) are at 110‐type positions (indexed according to the pseudo‐cubic unit cell), and there are none near 001‐type positions. Thus these diffraction patterns are on the 〈001〉 zone axis (electron beam parallel to the *z*‐axis) of the supposed L1_0_ FeNi. It would not be possible to measure the *c*/*a* ratio in this orientation using conventional electron diffraction. In the original work, the change in tetragonality measured as a function of temperature (Figure 5 in ref. [1]) cannot be due to the order‐disorder transition between tetrataenite and taenite. We conclude that there is no definitive and specific evidence for L1_0_ FeNi from electron microscopy in the present work or in ref. [1].

**Figure 5:**
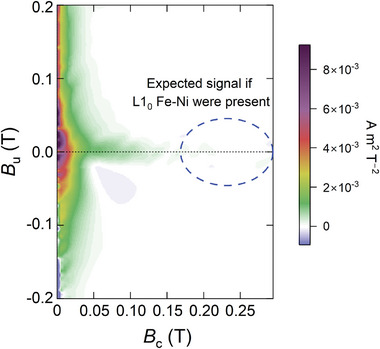
Magnetic characterization of as‐cast Fe_55_Ni_35_P_6.5_C_3.5._ The in‐plane first‐order reversal curve (FORC) diagram lacks any signal in the indicated region (dashed ellipse) of the high‐coercivity tail. Meteoritic samples containing tetrataenite do show a clear signal in this region.^[^ ^6^ ^]^

### 2.3 Magnetic Hysteresis

The original study^[^ ^1^ ^]^ did not quantify magnetic properties, but did contrast magnetic‐domain structures. The domains in primary dendrites within as‐cast Fe_55_Ni_35_P_6.5_C_3.5_ were larger than those in γ‐Fe_60_Ni_40_ suggesting higher coercivity. Characterizing the magnetic properties of multiphase materials is difficult; the complexity introduced by microstructure, domain state and the magnetic properties of each constituent phase means that the standard analysis of bulk hysteresis parameters can give ambiguous, misleading results.^[^ ^11^ ^]^ Quantitative analysis of magnetic mixtures is possible, however, with first‐order reversal curve (FORC) diagrams;^[^ ^12^ ^]^ notably these reveal the distribution of coercivity.^[^ ^11^ ^]^

Much effort has been devoted to the magnetic characterization of meteoritic samples;^[^ ^13^ ^]^ these are multi‐phase, and their microstructure varies from place to place. FORC diagrams indicate regions of high coercivity associated with single‐domain islands of tetrataenite.^[^ ^6^, ^7^ ^]^ The in‐plane FORC diagram for as‐cast Fe_55_Ni_35_P_6.5_C_3.5_ was determined and shows no evidence of high‐coercivity regions similar to those found in meteorites (Figure 5).

The coercivity of tetrataenite decreases with lower degrees of L1_0_ order,^[^ ^14^ ^]^ and decreased order could give a weak signal at 0.05‒0.15 T as seen in our FORC data (Figure 5). Such signals are, however, common in multi‐domain materials due to domain‐wall pinning by dislocations.^[^ ^15^ ^]^ We can rule out the presence of well ordered L1_0_ phase in our samples; it is more difficult to rule out a small volume fraction of poorly ordered L1_0_. Nevertheless, the weak FORC signals in the range 0.05–0.15 T, may be more readily explained by domain‐wall pinning. The FORC results (Figure 5) do, in any case, provide a baseline for future studies of related and optimized systems.

## 3 Discussion

The ordered L1_2_ FeNi_3_ phase is not found in the relatively iron‐rich alloys studied in the present work. Three phases are detected. The majority phase is γ‐(Fe,Ni) that forms as primary‐phase dendrites. Solidification is completed with formation of a eutectic mixture that includes (Fe,Ni)_3_P. On further cooling, α‐(Fe,Ni) is presumed to precipitate within the γ‐(Fe‐Ni) (Figure 1).

Importantly, the present measurements (X‐ray diffractograms, electron diffraction patterns, FORC diagrams) do not provide any evidence for the presence of tetrataenite in as‐cast Fe_55_Ni_35_P_6.5_C_3.5_ (the same sample as studied in the original study^[^ ^1^ ^]^) or in as‐cast (Fe_55_Ni_45_)_94_P_6_. We conclude that the observations in ref. [1] need to be re‐interpreted, highlighting the difficulties in achieving secure identification of tetrataenite. The present electron‐diffraction work, for example, shows that 311‐type reflections from Fe_3_O_4_ (Table 1) can be misinterpreted as 110‐type reflections from tetrataenite (indexed according to the pseudo‐cubic unit cell). The possibility of this misinterpretation was previously highlighted by Reuter et al.^[^ ^10^ ^]^ In general, it is difficult to detect chemical ordering to tetrataenite using diffraction methods,^[^ ^16^ ^]^ and the present work provides a ‘warning sign’ for future studies of that ordering.

The presence of phosphorus in Fe‐Ni alloys does increase atomic diffusivities^[^ ^17^ ^]^ and, for example accelerates the growth of kamacite in meteoritic taenite.^[^ ^18^ ^]^ As pointed out in the original work,^[^ ^1^ ^]^ the presence of phosphorus should be taken into account in any further analysis of the kinetics of tetrataenite formation in meteorites. Unfortunately, the present work does not support the hope that higher phosphorus contents could so accelerate diffusivities as to permit the formation of tetrataenite in conventionally cast samples. Moreover, the present work shows that the presence of (Fe,Ni)_3_P in samples can contribute (in XRD results) to an impression that tetrataenite is present.

It remains a high priority to find a hard‐magnetic material capable of competing with Nd‐Fe‐B. A key aim is to avoid the use of rare earths, considered to present a supply risk^[^ ^19^ ^]^ and requiring extraction that is detrimental to the environment. Tetrataenite is prominent among those materials considered as candidates for rare‐earth‐free permanent magnets.^[^ ^20^ ^]^ Its magnetic hardness *κ* cannot match that of Nd‐Fe‐B,^[^ ^21^ ^]^ yet with low‐enough price and low environmental impact there might still be a market opportunity. Can the ordering to achieve tetrataenite be achieved in timescales practicable for production of magnets? While the presence of phosphorus may assist in reaching the required ordering rates, it is not sufficient. Severe plastic deformation (SPD) also increases atomic mobilities and, combined with annealing, has been tried as a route to tetrataenite formation.^[^ ^22^ ^]^ Annealing under combined applied stress and magnetic field does induce L1_0_‐type ordering in equiatomic FeNi.^[^ ^16^ ^]^ A priority for future studies is to apply processing of these kinds to phosphorus‐containing alloys.

Even if synthesis of tetrataenite can be achieved on reasonable timescales, studies of meteorites suggest that the best hard‐magnetic performance can be achieved only if the regions of tetrataenite are sufficiently small to be single‐domain.^[^ ^7^ ^]^ This is a further barrier, yet to be addressed, in manufacture of industrially competitive permanent magnets based on tetrataenite.

## 4 Conclusion

Recent work^[^ ^1^ ^]^ suggested that conventional casting of Fe‐Ni‐P‐(C) alloys can give samples with a significant volume fraction of L1_0_ FeNi tetrataenite, a phase found in meteorites that has the potential to be the basis of a rare‐earth‐free permanent‐magnet material. Following directly from that work, the present study has investigated this claim, reanalyzing the original data and acquiring new data using X‐ray diffraction, electron diffraction and magnetometry.

Tetrataenite would be formed by chemical ordering within γ‐(Fe,Ni), giving a slight tetragonal distortion. The XRD Bragg peaks formerly taken to reveal that distortion are now attributed to γ‐(Fe,Ni) and (Fe,Ni)_3_P. Electron diffraction patterns in the original work appeared to show superlattice reflections indicating the presence of tetrataenite. These apparent reflections arise from an oxide layer caused by the plasma‐cleaning that was part of the thin‐foil preparation; this highlights the difficulties in characterizing the chemical ordering in the Fe‐Ni solid solution, We have made a preliminary characterization of the distribution of magnetic coercivity in as‐cast Fe‐Ni‐P‐(C) alloys; this does not reveal the presence of any hard‐magnetic phase. Although the presence of phosphorus in these alloys has the potential to accelerate the ordering required to form tetrataenite, there is no evidence for any presence of tetrataenite in as‐cast samples, and ref. [1] is retracted.

## 5 Experimental Section

### Sample Preparation

A 3‐mm‐diam rod of (Fe_55_Ni_45_)_94_P_6_ (at.%) was prepared similarly to those in ref. [1] Further studies were conducted on an as‐cast sample of Fe_55_Ni_35_P_6.5_C_3.5_ from the previous work.^[^ ^1^ ^]^

### X‐Ray Diffraction

Slices were cut from suction‐cast rods. Both as‐cast and annealed slices were mechanically polished with diamond paste to a 1‐µm mirror finish to remove potential surface effects, including oxidation. X‐ray diffraction of as‐cast and annealed samples was performed using a Bruker D8 Advance diffractometer combined with a LynxEye position‐sensitive detector. As in previous work,^[^ ^1^ ^]^ CoKα radiation (*λ*_Kα1_  = 1.7890 Å; *λ*_Kα2_  = 1.7928 Å) was used.

### Transmission Electron Microscopy

An FEI NanoLab‐600 Helios Dual‐Beam focused ion beam (FIB) microscope equipped with an Omniprobe‐200 micromanipulator was used to prepare samples for TEM. Gallium ion milling (30 kV, 90 pA–1.4 nA) was used to cut lamellae ≈5 × 20 µm in area. These were extracted from the bulk sample using a micromanipulator. Platinum deposition (approx. 100–200 nm thick) was used to mount the lamellae on Omniprobe copper grids before they were finally milled to an electron‐transparent thickness of 50–100 nm. Plasma‐cleaning was carried out using a Fischione model 1020. In this cleaner, a 1:3 mixture of oxygen and argon gas is excited by an electromagnetic field oscillating at 13.56 MHz to create the plasma, from which ions impinge upon the specimen with energies of lower than 12 eV. The cleaning is intended to oxidize and remove carbonaceous material contaminating the surfaces of the thin foil.^[^ ^23^ ^]^ The thin foils were characterized using TEM both before and after the plasma‐cleaning step.

TEM was conducted using an FEI Tecnai F20 equipped with a field‐emission electron gun operated at an accelerating voltage of 200 kV. Images and diffraction patterns were recorded using a Gatan OneView camera with 4096 × 4096 pixels. The specimens were investigated at room temperature using a Thermo‐Fisher double‐tilt specimen holder (model number 5322 695 15 872) and heated in situ using a Philips PW6592 single‐tilt heating holder.

### Vibrating Sample Magnetometry

Magnetic measurements were made on a (Fe_55_Ni_45_)_94_P_6_ sample using a Lake Shore Cryotronics 8600 series room‐temperature vibrating‐sample magnetometer (VSM). The sample was a plate cut from the as‐cast rod, and measurements were made in‐plane and out‐of‐plane. First‐order reversal curve (FORC) diagrams were determined as in ref. [12] and analyzed using *FORCinel* software.^[^ ^24^ ^]^

## Conflict of Interest

The authors declare no conflict of interest.
